# Biological variation estimates of Alzheimer's disease plasma biomarkers in healthy individuals

**DOI:** 10.1002/alz.13518

**Published:** 2023-11-20

**Authors:** Wagner S. Brum, Nicholas J. Ashton, Joel Simrén, Guiglielmo di Molfetta, Thomas K. Karikari, Andrea L. Benedet, Eduardo R. Zimmer, Juan Lantero‐Rodriguez, Laia Montoliu‐Gaya, Andreas Jeromin, Aasne K. Aarsand, William A. Bartlett, Pilar Fernández Calle, Abdurrahman Coşkun, Jorge Díaz–Garzón, Niels Jonker, Henrik Zetterberg, Sverre Sandberg, Anna Carobene, Kaj Blennow

**Affiliations:** ^1^ Department of Psychiatry and Neurochemistry, Institute of Neuroscience and Physiology The Sahlgrenska Academy at the University of Gothenburg Mölndal Sweden; ^2^ Department of Biochemistry Universidade Federal do Rio Grande do Sul (UFRGS) Porto Alegre Brazil; ^3^ King's College London, Institute of Psychiatry Psychology and Neuroscience Maurice Wohl Institute Clinical Neuroscience Institute London UK; ^4^ NIHR Biomedical Research Centre for Mental Health and Biomedical Research Unit for Dementia at South London and Maudsley NHS Foundation London UK; ^5^ Centre for Age‐Related Medicine Stavanger University Hospital Stavanger Norway; ^6^ Clinical Neurochemistry Laboratory Sahlgrenska University Hospital Mölndal Sweden; ^7^ Department of Psychiatry University of Pittsburgh Pittsburgh Pennsylvania USA; ^8^ Department of Pharmacology Universidade Federal do Rio Grande do Sul (UFRGS) Porto Alegre Brazil; ^9^ Graduate Program in Biological Sciences Universidade Federal do Rio Grande do Sul (UFRGS) Porto Alegre Brazil; ^10^ McGill Centre for Studies in Aging McGill University Verdun Quebec Canada; ^11^ ALZpath. Inc Carlsbad California USA; ^12^ European Federation of Clinical Chemistry and Laboratory Medicine Working Group on Biological Variation Milan Italy; ^13^ The Norwegian Organization for Quality Improvement of Laboratory Examinations (NOKLUS) Haraldsplass Deaconess Hospital Bergen Norway; ^14^ School of Science and Engineering University of Dundee Dundee UK; ^15^ Department of Laboratory Medicine La Paz University Hospital Madrid Spain; ^16^ School of Medicine, Department of Medical Biochemistry Acibadem Mehmet Ali Aydınlar University Istanbul Turkey; ^17^ Certe Wilhelmina Ziekenhuis Assen Assen the Netherlands; ^18^ Department of Neurodegenerative Disease UCL Institute of Neurology London UK; ^19^ UK Dementia Research Institute at UCL London UK; ^20^ Hong Kong Center for Neurodegenerative Diseases Hong Kong China; ^21^ Wisconsin Alzheimer's Disease Research Center, School of Medicine and Public Health University of Wisconsin–Madison Madison Wisconsin USA; ^22^ Department of Global Health and Primary Care, Faculty of Medicine University of Bergen Bergen Norway; ^23^ Laboratory Medicine IRCCS San Raffaele Scientific Institute Milan Italy

**Keywords:** amyloid, analytical variation, biological variation, glial fibrillary acidic protein, neurofilament light, plasma biomarkers, phosphorylated tau, reference change values

## Abstract

**INTRODUCTION:**

Blood biomarkers have proven useful in Alzheimer's disease (AD) research. However, little is known about their biological variation (BV), which improves the interpretation of individual‐level data.

**METHODS:**

We measured plasma amyloid beta (Aβ42, Aβ40), phosphorylated tau (p‐tau181, p‐tau217, p‐tau231), glial fibrillary acidic protein (GFAP), and neurofilament light chain (NfL) in plasma samples collected weekly over 10 weeks from 20 participants aged 40 to 60 years from the European Biological Variation Study. We estimated within‐ (CV_I_) and between‐subject (CV_G_) BV, analytical variation, and reference change values (RCV).

**RESULTS:**

Biomarkers presented considerable variability in CV_I_ and CV_G_. Aβ42/Aβ40 had the lowest CV_I_ (≈ 3%) and p‐tau181 the highest (≈ 16%), while others ranged from 6% to 10%. Most RCVs ranged from 20% to 30% (decrease) and 25% to 40% (increase).

**DISCUSSION:**

BV estimates for AD plasma biomarkers can potentially refine their clinical and research interpretation. RCVs might be useful for detecting significant changes between serial measurements when monitoring early disease progression or interventions.

**Highlights**

- Plasma amyloid beta (Aβ42/Aβ40) presents the lowest between‐ and within‐subject biological variation, but also changes the least in Alzheimer's disease (AD) patients versus controls.
- Plasma phosphorylated tau variants significantly vary in their within‐subject biological variation, but their substantial fold‐changes in AD likely limits the impact of their variability.
- Plasma neurofilament light chain and glial fibrillary acidic protein demonstrate high between‐subject variation, the impact of which will depend on clinical context.
- Reference change values can potentially be useful in monitoring early disease progression and the safety/efficacy of interventions on an individual level.
- Serial sampling revealed that unexpectedly high values in heathy individuals can be observed, which urges caution when interpreting AD plasma biomarkers based on a single test result.

## 1 BACKGROUND

Novel technologies to measure brain pathophysiological processes in the blood have revolutionized the Alzheimer's disease (AD) research landscape.^1^, ^2^ Established and highly accurate methods for tracking such processes face barriers to their large‐scale implementation, such as the high costs, radiation exposure, and limited availability of positron emission tomography (PET) scans, as well as the relative invasiveness of lumbar punctures, required for measuring AD biomarkers in the cerebrospinal fluid (CSF).^3^ Blood‐based AD biomarkers have demonstrated great promise so far, and are particularly promising for scalable implementation due to their minimally invasive and cost‐effective nature.^1^, ^2^

Among blood‐based biomarkers so far investigated, plasma phosphorylated tau (p‐tau) variants, such as p‐tau181, p‐tau231, and p‐tau217, have demonstrated the greatest potential to identify AD‐specific processes, showing high accuracy for identifying neuropathological or biomarker‐confirmed AD and predicting cognitive decline.^4^, ^5^, ^6^, ^7^, ^8^ While p‐tau231 may be more sensitive to incipient amyloid beta (Aβ) pathology, plasma p‐tau217 seems the most well suited for clinical implementation, presenting the highest fold increases in cognitively impaired patients with AD‐type pathology, and it can dynamically track longitudinal AD clinical progression.^4^, ^6^, ^7^, ^9^, ^10^, ^11^ Plasma Aβ, in the form of the Aβ42/Aβ40 ratio, has also shown good performance in detecting Aβ pathology, but its modest fold change (reduced by 8% to 14% in AD compared to Aβ‐negative controls, when in the CSF it is reduced by > 50%)^12^, ^13^ makes it more vulnerable to analytical fluctuations normally observed in a day‐to‐day clinical chemistry routine.^14^, ^15^, ^16^ Plasma levels of glial fibrillary acidic protein (GFAP), a cytoskeletal protein highly expressed in reactive astrocytes,^17^ have been positively associated with early Aβ pathology.^18^, ^19^, ^20^, ^21^ Neurofilament light chain (NfL), a marker for axonal damage, has gained increasingly clinical significance with robust evidence for its diagnostic and prognostic utility in a wide range of neurodegenerative diseases (AD, frontotemporal dementia, atypical parkinsonian disorders) and in acute neurological conditions, such as stroke and traumatic brain injury.^22^, ^23^, ^24^, ^25^, ^26^ Furthermore, all of these biomarker candidates have been evaluated as potential surrogate endpoints in disease‐modifying clinical trials in AD, with a recent example being reductions in plasma p‐tau217 as early as after 12 weeks of treatment with a promising anti‐Aβ monoclonal antibody, donanemab.^27^

**RESEARCH IN CONTEXT**

1. **Systematic review**: We reviewed PubMed for articles and conference abstracts that evaluated the biological variation (BV) of novel Alzheimer's disease (AD) blood biomarkers. Two previous studies had reported BV estimates for serum glial fibrillary acidic protein (GFAP) and neurofilament light chain (NfL). Thus, we aimed to provide the first robust BV estimates for plasma amyloid beta (Aβ) and phosphorylated tau (p‐tau) biomarkers, as well as for plasma GFAP and NfL in in the same population.
2. **Interpretation**: Plasma biomarkers of key pathological features of AD demonstrate heterogeneity in their within‐ and between‐subject variation. Plasma Aβ42/Aβ40 generally shows lower variability but also changes very modestly in AD patients versus controls. While plasma p‐tau variants demonstrate higher variability, the clinical impact is likely limited due to large fold increases in AD. Plasma NfL and GFAP had the largest between‐subject variability, which may impact their application in certain contexts. Most research on blood biomarkers so far has been done using either single measurements or repeated measurements over longer (e.g., yearly) time frames; the weekly serial sampling in our study revealed that unexpected outlier values may occur, urging caution in clinical and research interpretation.
3. **Future directions**: Future studies should evaluate the potential clinical impact of the application of BV knowledge upon clinical and research interpretation of AD plasma biomarkers, especially in disease monitoring and in the evaluation of safety and efficacy of novel therapeutic interventions.

Nevertheless, several research questions must be addressed before large‐scale implementation of blood‐based AD biomarkers.^28^ While most studies have focused on their diagnostic and prognostic properties, little is known about their biological variation (BV), a foundational concept in clinical chemistry, crucial to ensure the safe implementation of diagnostic markers and to minimize misclassification risks in laboratory medicine.^29^ BV refers to the variation observed in clinical laboratory measurements determined by patients’ physiology, and a strict guideline‐defined methodology must be followed by BV studies to ensure robust results.^30^, ^31^ Such studies require the serial, tightly controlled collection of samples from healthy individuals with a regular sampling rate, and that analytes should be quantified, at least, in duplicate.^30^, ^31^ The key BV components are the within‐subject biological variation (CV_I_), which informs how much the concentration of a biomarker fluctuates around each individual's homeostatic setpoint, and the between‐subject biological variation (CV_G_), which informs on the variability between the homeostatic setpoints between different individuals. These parameters, alongside known assay‐dependent analytical variation (CV_A_), can provide highly clinically useful information for biomarker implementation. These include the reference change value (RCV),^32^, ^33^ which enumerates the change needed between consecutive measurements to exceed biological and analytical variation; the analytical performance specifications (APS) that clinical‐grade assays should meet;^34^, ^35^ and the index of individuality (II), which evaluates the utility of population based reference intervals.^33^, ^36^ Thus, high‐quality BV data are needed in this rapidly developing area of AD diagnostics, in which specific biomarkers and assays are being considered for clinical implementation and therapeutic trial use.

Here, we aimed to determine BV estimates for plasma Aβ42, Aβ40, Aβ42/Aβ40, p‐tau181, p‐tau217, p‐tau231, GFAP, and NfL (and associated APS and RCVs) in healthy adults between 40 and 60 years from the European Biological Variation Study (EuBIVAS), led by the European Federation of Clinical Chemistry and Laboratory Medicine (EFLM) Working Group on Biological Variation.^37^, ^38^ The EuBIVAS is a highly powered multi‐center study that included weekly blood sampling over 10 weeks from presumably healthy volunteers from five European countries and that has delivered high‐quality BV estimates for many measurands widely used in diverse medical areas.^38^, ^39^, ^40^, ^41^, ^42^

## 2 METHODS

### 2.1 Study participants and sample collection

In this study, we quantified biomarkers in plasma‐citrate samples from a subset of 20 individuals aged between 40 and 60 years within the EuBIVAS,^37^, ^38^ which originally enrolled 91 healthy volunteers (53 females, 38 males; ages 21 to 69 years), from six European laboratories located in five different countries (Italy, Norway, Spain, Turkey and the Netherlands). We chose to include in the current study those in the older EuBIVAS age range that had sufficient sample material for analyses. Information on the participants’ health status and lifestyle was collected with an enrollment questionnaire, and participants were screened at enrollment with a selection of laboratory tests to further confirm compatibility with inclusion criteria. Fasting blood samples were collected weekly over 10 consecutive weeks for each study participant (April–June 2015), always in the morning. At each center, samples were centrifuged at 3000 × g for 10 minutes at room temperature within 1 hour of the blood draw, aliquoted, and frozen rapidly by immersion in a bowl with methanol and dry ice, and sent to the coordinating center (San Raffaele Hospital in Milan, Italy), where they were stored at −80°C. In November 2021, the samples included in this study were sent to the Clinical Neurochemistry Laboratory (Sahlgrenska University Hospital, Gothenburg, Sweden), where the AD blood biomarkers were measured (April 2022, except p‐tau217, analyzed December 2022). Further details regarding the inclusion/exclusion criteria; health status; and sample collection, processing, and storage protocol used in EuBIVAS have been previously reported.^37^ While there was a considerable gap between sample collection and biochemical analyses (≈ 7 years), the evaluated AD blood biomarkers are known to be stable under the storage conditions used for the samples tested in this project.^4^, ^5^, ^18^, ^23^

The protocol for EuBIVAS received approval from the institutional ethical review board of San Raffaele Hospital, in compliance with the World Medical Association Declaration of Helsinki, as well as the ethical board/regional ethics committee for each participating center.

### 2.2 Biomarker quantification

Biomarker quantification was conducted using single molecule array (Simoa) HD‐X Analyzers from Quanterix at the Clinical Neurochemistry Laboratory of Sahlgrenska University Hospital in Sweden. A commercially available assay (Quanterix Neurology‐4 Plex E) was used to simultaneously quantify for Aβ42, Aβ40 (and Aβ42/Aβ40, consequently), NfL, and GFAP.^27^ P‐tau231 and p‐tau181 were analyzed using Simoa assays developed at the University of Gothenburg, which have been validated as described elsewhere.^5^, ^6^ To measure p‐tau217, a novel commercially available assay from ALZpath (ALZpathDX) was used.^43^ All samples from the same participant were analyzed in the same analytical run, and each sample was quantified in duplicate. Internal quality controls (iQC) at three different concentrations, for each measurand, were analyzed in duplicate in the beginning and end of each run. Before analysis, blood samples were thawed, vortexed, and centrifuged at 4000 × g for 10 minutes as suggested in recent studies.^44^, ^45^

### 2.3 Statistical analysis

Our statistical analyses followed a series of well‐established and guideline‐defined steps for deriving BV data, as set out by the Biological Variation Data Critical Appraisal Checklist (BIVAC), a standard for the executing and reporting of BV studies.^31^ Outlier detection procedures were performed on three levels, including analytical (between replicates), within‐subject (among 10 collections for CV_I_ calculation), and between‐subject level (for CV_G_ calculation).^46^, ^47^, ^48^, ^49^, ^50^ For obtaining CV_I_ and CV_A_ estimates, we initially performed CV‐transformation of the data in which each person's data are “normalized” by dividing by that person's mean value, so as to later perform analysis of variance (ANOVA) on these CV‐transformed values.^51^ After CV transformation, we performed outlier identification and removal on the analytical levels (between replicates) by assessing the homogeneity of CV_A_ with the Bartlett test. In case of heterogeneity for the analytical component, we first excluded the replicate value of the measurement that most deviated from that participant's mean. If the heterogeneity persisted, we then also excluded the second measurement result of the time point showing abnormal analytical variation. After ensuring analytical homogeneity, we evaluated the presence of outliers on the within‐individual variation level by assessing the homogeneity of the within‐individual CV_I_ with the Cochran test. Then, we evaluated for each biomarker whether the results were consistent with steady state (i.e., no trend for increase or decrease during study) by fitting a linear regression model with the mean blood drawing value (pooled mean of the duplicate concentration measurements of each participant) as the dependent variable, with blood drawing number (from 1 to 10) as the independent variable. Individuals were considered in a steady state if the 95% confidence interval (CI) of the blood draw term (i.e., the slope) included 0.^52^ Finally, the CV_I_ was estimated with CV‐ANOVA, the “Røraas method,” a validated and recommended ANOVA method for estimating CV_I_ and CV_A_.^51^, ^53^ To calculate the between‐subject biological variation (CV_G_), we first applied the Dixon *Q* test to detect outliers in mean biomarker concentrations between subjects, and the Shapiro–Wilk test to verify the normality assumption on mean concentrations. If the latter tests detected a non‐normal distribution, concentration data were natural log‐transformed, prior to obtaining the CV_G_ by ANOVA.^46^, ^47^, ^48^, ^49^, ^50^ First, CV_I_ and CV_G_ estimates were calculated for the whole study population, and also secondarily separately for males and females, for all measurands. Confidence intervals for BV estimates were calculated as previously described,^54^ and the lack of overlap of the 95% CI of estimates was used to indicate significant differences between subgroups.

Other relevant metrics were computed based on the above‐mentioned BV estimates calculated as follows. Desirable APS were calculated for imprecision (CV_APS_ = 0.5_x_CV_I_) and for bias (Bias_APS_ = 0.25_x_√[CV_I_^2^ + CV_G_^2^]). The RCV was calculated at a 95% bidirectional alpha (z = 1.65) as RCV = 100*(exp[± z_x_√2_x_σ]–1), where σ = √ln(σ^2^_CVi_ + σ^2^_CVi_), with σ^2^_CVi_ = ln(CVi^2^ + 1) and σ^2^_CVa_ = ln(CVa^2^ + 1). The II was calculated as the ratio of CV_I_ and CV_G_ for each biomarker, indicating whether population‐based reference intervals can be useful for evaluating results.^33^, ^36^ We also calculated the number of samples needed to be collected to estimate an individual's homeostatic point (NHSP) with a “D” absolute percentage proximity to the individual's true value with the equation *n* = (z_x_√[CV_I_^2^ + CV_A_^2^]/D)^2^, in which z = 1.96, corresponding to a 95% alpha. NHSP was calculated based on 5%, 10%, and 20% deviations from the homeostatic setpoint. Metrics such as RCVs and APS were always derived based on CV_I_ and CV_G_ of all participants. All analyses were performed with R Statistical Software (version 4.2.1; www.r‐project.com), and statistical significance was set as alpha = 0.05.

## 3 RESULTS

### 3.1 Participant characteristics

We included data analyzed from a total of 196 plasma samples, collected weekly over 10 weeks from 20 participants, with a mean number of 9.8 samples per participant. Key demographic information is described in Table 1. The age range of the included participants was 40 to 60 years, with a mean (standard deviation [SD]) age of 46.4 years (6.20) for the whole study population. Half of the participants were female, and key demographic characteristics were generally similar between sexes. The study population came from five centers in four European countries (Italy [*n* = 7], Netherlands [*n* = 5], Norway [*n* = 5], Spain [*n* = 3]), and all participants were White. Participants were healthy, with a mean (SD) body mass index of 23.3 kg/m^2^ (2.85 kg/m^2^), did not have hypertension, and the majority (55%) engaged in physical activity for more than 3 hours per week. Only one participant was a smoker (5%), and 11 reported consuming 1 to 2 units of alcohol per week.[Table alz13518-tbl-0002]

**TABLE 1:** Demographic characteristics.

	Female (*n* = 10)	Male (*N* = 10)	Overall (*N* = 20)
Age, years, mean (SD)	47.0 (5.98)	45.8 (6.68)	46.4 (6.20)
BMI, kg/m^2^, mean (SD)	22.3 (2.50)	24.2 (2.98)	23.3 (2.85)
Hypertension, *n* (%)	0	0	0
Alcohol consumption, units/week, *n* (%)			
0	3 (30.0)	3 (30.0)	6 (30.0)
1–2	6 (60.0)	5 (50.0)	11 (55.0)
≥ 3	1 (10.0)	2 (20.0)	4 (15.0)
Smokers, *n* (%)	1 (10)	0	1 (5)
Physical exercise, *n* (%)	8 (80)	5 (50)	13 (65)
No physical exercise	2 (20)	5 (50)	7 (35)
< 3 hours per week	1 (10)	1 (10)	2 (10)
≥ 3 hours per week	7 (70)	4 (40)	11 (55)
Study center, *n* (%)			
Italy (Milan)	2 (20)	2 (20)	4 (20)
Italy (Padua)	3 (30)	0	3 (15)
Netherlands	3 (30)	2 (20)	5 (25)
Norway	2 (20)	3 (30)	5 (25)
Spain	0	3 (30)	3 (15)

*Note*: The table summarizes key demographic information for the included participants. Data are described as mean (SD) or *n* (%).

Abbreviations: BMI, body mass index; SD, standard deviation.

### 3.2 Homogeneity analyses and outliers

Table 2 displays results of the homogeneity analyses for outlier detection and the final number of results included for each of the biomarkers. All samples were always analyzed in duplicate, except in very few cases with insufficient volume left, resulting in a mean of 1.97 replicate quantification per sample per biomarker. When evaluating the analytical homogeneity with the Bartlett test, no outliers for the replicate measurement were identified for Aβ40, Aβ42/Aβ40, GFAP, and NfL, while a few replicates were excluded for Aβ42, p‐tau181, p‐tau217, and p‐tau231. When assessing the variance homogeneity for within‐subject variation, outlier time points were identified for all biomarkers, but no subject had to be fully excluded. For the total study population, the mean percentage of results identified as outliers at the homogeneity analyses was 3.56% (range, 1.0% to 6.8%), which left a mean of 369 results (range, 332 to 381) used per biomarker to estimate the CV_I_.

**TABLE 2 alz13518-tbl-0002:** Homogeneity analyses and number of results included for calculation of biological variation estimates.

					Number of excluded results/subjects					
					Homogeneity (Bartlett and Cochran tests)			Dixon *Q* test		
Biomarker	Subjects, *n*	Total measurements, *n*	Mean number of samples/ individual	Mean number of replicates/ sample	Replicates (analytical homogeneity)	Samples (within homogeneity)	Subjects (within homogeneity)	Subjects (between)	Data used to estimate CV_I_, *n*	Total % of outliers
Aβ40	20	385	9.80	1.96	0	8	0	0	377	2.08%
Aβ42	20	385	9.80	1.96	2	8	0	0	375	2.60%
Aβ42/Aβ40	20	385	9.80	1.96	0	4	0	2	381	1.04%
GFAP	20	385	9.80	1.96	0	7	0	0	378	1.82%
NfL	20	385	9.80	1.96	0	8	0	1	377	2.08%
P‐tau181	20	392	9.80	2.00	4	18	0	0	370	5.61%
P‐tau217	20	355	9.45	1.88	9	14	0	0	332	6.48%
P‐tau231	20	384	9.60	2.00	4	22	0	0	358	6.77%

*Note*: The table displays the overall number of samples included and biomarker results produced, as well as the results of the homogeneity analyses carried to detect the presence of outliers on the replicate, sample, and subject levels.

Abbreviations: Aβ, amyloid beta; CV_I_, within‐individual biological variation; GFAP, glial fibrillary acidic protein; NfL, neurofilament light; p‐tau, phosphorylated tau.

**FIGURE 1:**
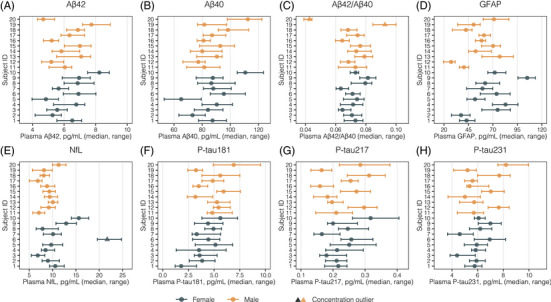
Participant‐level plasma biomarker concentrations over 10 weeks. The figure displays median (dots) and range (error bars) of biomarker concentrations over 10 weeks. Females are represented in dark green, and males in orange, and participants are shown with increasing age (subject 1 is the youngest female participant, and subject 20 the oldest male). Triangles represent the concentration outliers detected with the Dixon *Q* test before the CV_G_ calculation. Aβ, amyloid beta; CV_G_, between‐subject biological variation; GFAP, glial fibrillary acidic protein; NfL, neurofilament light; p‐tau, phosphorylated tau.

In Figure 1 the 10‐week biological variation, in concentrations, of each plasma biomarker, stratified by sex and ordered by increasing age, is displayed. In a separate outlier detection procedure before the CV_G_ estimation, the Dixon *Q* test identified one outlier subject for NfL, and two outlier subjects for Aβ42/Aβ40 (indicated in Figure 1). No trend was identified for any of the included biomarkers in the overall study population or in male or female subgroups. No biomarker measurement for any analyte was below the lower limit of detection or the lower limit of quantification.

### 3.3 Analytical performance (CV_A_)

**FIGURE 2:**
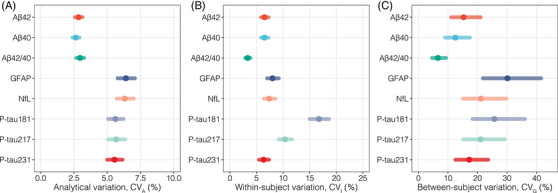
Biological variation estimates in the whole study population. The forest plot graphically summarizes the biological variation estimates obtained in this study, with dots corresponding to point estimates and error bars to 95% CIs. A, Analytical variation (CV_A_), (B) estimates for within‐individual biological variation, (C) between‐individual biological variation. Aβ, amyloid beta; CI, confidence interval; CV_A_, analytical variation; CV_G_, between‐subject biological variation; CV_I_, within‐subject biological variation; GFAP, glial fibrillary acidic protein; NfL, neurofilament light; p‐tau, phosphorylated tau.

The CV_A_ for each biomarker, which indicates the imprecision between duplicate measurements, and associated 95% CIs, are graphically displayed in Figure 2A and numerically represented in Table 3. The CV_A_ ranged from ≈ 3% for all Aβ biomarkers (Aβ42: 2.8%; Aβ40: 2.6%; Aβ42/Aβ40: 3.0%), to ≈ 6% for GFAP (6.4%) and NfL (6.3%), and to ≈ 5.5% for all p‐tau biomarkers (p‐tau181: 5.6%; p‐tau217: 5.7%; p‐tau231: 5.6%). Analytical variability of internal quality controls presented similar CVs to those estimated with CV‐ANOVA, and no systematic trends in concentration change between runs were observed by visual inspection.

**TABLE 3:** Biological variation estimates for the whole study population and according to sex.

Biomarker	Study population	Mean concentration, (pg/mL*, 95% CI)	CV_A_ (%, 95% CI)	CV_I_ (%, 95% CI)	CV_G_, (%, 95% CI)
Aβ42	All participants	6.24 (6.13–6.35)	2.84 (2.57–3.15)	6.50 (5.82–7.27)	15.3 (11.1–21.1)
	Male	6.21 (6.06–6.36)		6.26 (5.34–7.35)	15.6 (9.56–25.4)
	Female	6.26 (6.11–6.42)		6.76 (5.79–7.89)	15.8 (9.97–25.1)
Aβ40	All participants	88.0 (86.8–89.3)	2.63 (2.38–2.91)	6.39 (5.73–7.13)	12.5 (9.03–17.4)
	Male	88.9 (87.2–90.6)		6.26 (5.36–7.31)	11.6 (7.18‐18.7)
	Female	87.2 (85.3–89.2)		6.76 (5.80–7.88)	14.1 (8.76–22.6)
Aβ42/Aβ40	All participants	0.0714 (0.0704–0.0724)	2.95 (2.67–3.26)	3.33 (2.88–3.85)	6.58 (4.64–9.34)
	Male	0.0707 (0.0689–0.0726)		3.54 (2.90–4.32)	6.22 (3.59–10.8)
	Female	0.0720 (0.0712–0.0729)		3.13 (2.53–3.88)	7.20 (4.47–11.6)
GFAP	All participants	59.9 (58–61.7)	6.40 (5.78–7.08)	8.01 (6.99–9.18)	30.1 (21.8–41.6)
	Male	54.9 (52.6–57.2)		8.12 (6.74–9.77)	28.5 (17.8–45.5)
	Female	64.9 (62.2–67.7)		7.95 (6.5–9.72)	29.7 (18.6–47.5)
NfL	All participants	10 (9.7–10.3)	6.32 (5.71–7.00)	7.39 (6.4–8.52)	21.2 (15.1–29.8)
	Male	8.8 (8.6–9.1)		7.16 (5.89–8.71)	15.9 (9.8–25.8)
	Female	11.2 (10.6–11.8)		7.67 (6.23–9.44)	24.9 (15.0–42.1)
P‐tau181	All participants	4.56 (4.41–4.70)	5.62 (5.07–6.23)	16.7 (15.0–18.6)	25.7 (18.3–36.1)
	Male	4.97 (4.77–5.18)		13.3 (11.4–15.5)	24.8 (15.3–40.1)
	Female	4.13 (3.95–4.31)		19.7 (16.9–22.9)	23.7 (14.0–40.4)
P‐tau217	All participants	0.232 (0.226–0.238)	5.67 (5.07–6.35)	10.3 (9.15–11.7)	21.1 (15.1–29.3)
	Male	0.236 (0.226–0.245)		10.5 (8.92–12.4)	24.8 (15.4–39.9)
	Female	0.229 (0.222–0.236)		10.3 (8.58–12.3)	17.9 (11.0–29.0)
P‐tau231	All participants	6.06 (5.94–6.18)	5.55 (5.0–6.15)	6.33 (5.46–7.35)	17.2 (12.5–23.6)
	Male	6.31 (6.12–6.5)		5.49 (4.32–6.98)	19.7 (12.3–31.4)
	Female	5.82 (5.68–5.95)		7.1 (5.86–8.61)	13.9 (8.63–22.4)

*Note*: The table displays the biological variation estimates for each biomarker and mean concentrations for the whole participant population in sex‐stratified sub‐groups and their 95% CIs.

Abbreviations: Aβ, amyloid beta; CI, confidence interval; CV_A_, analytical variation; CV_G_, between‐individual biological variation; CV_I_, within‐individual biological variation; GFAP, glial fibrillary acidic protein; NfL, neurofilament light; p‐tau, phosphorylated tau.

### 3.4 Within‐subject biological variation (CV_I_)

Figure 2B graphically represents the CV_I_ values and their associated 95% CIs, that is, how much biomarker concentrations fluctuate around each individual's homeostatic setpoint. Plasma Aβ42 and Aβ40 demonstrated low and very similar CV_I_’=s (Aβ42: 6.5%, 95% CI: 5.8–7.3; Aβ40: 6.4%, 95% CI: 5.7–7.1), and the plasma Aβ42/Aβ40 ratio demonstrated the lowest CV_I_ among all evaluated biomarkers (3.3%, 95% CI: 2.9–3.9). Among plasma p‐tau variants, p‐tau231 demonstrated the lowest CV_I_ (6.3%, 95% CI: 5.5–7.4), followed by p‐tau217 (10.3%, 95% CI: 9.2–11.7), and by p‐tau181 with a considerably higher CV_I_ (16.7%, 95% CI: 15.0–18.6). Plasma GFAP also demonstrated a relatively low CV_I_ (8.0%, 95% CI: 7.0–9.2), comparable to that observed for NfL (7.4%, 95% CI: 6.4–8.5). In Table 3, the CV_I_s are also shown separately for males and females, important and needed subgroup analyses in BV studies. Except for p‐tau181, no differences in CV_I_ were observed for the evaluated biomarkers, with overlapping 95% CIs for male and female CV_I_s. For plasma p‐tau181, females (19.7%, 95% CI: 16.9–22.9) demonstrated a higher CV_I_ than males (13.3%, 95% CI: 11.4–15.5).

### 3.5 Between‐subject biological variation (CV_G_)

Figure 2C graphically represents the CV_G_ values and their associated 95% CIs, that is, how much biomarker levels vary among healthy individuals. Among Aβ biomarkers, plasma Aβ42/Aβ40 demonstrated the lowest CV_G_ (6.6%, 95% CI: 4.6–9.3), with higher and similar estimates for Aβ42 (15.3%, 95% CI: 11.1–21.1) and Aβ40 (12.5%, 95% CI: 9.0–17.4). For the other biomarkers, CV_G_s were generally higher than those for Aβ biomarkers. GFAP demonstrated the highest CV_G_ among all biomarkers (30.1%, 95% CI: 21.8–41.6), and slightly higher than that of NfL (21.2%, 95% CI: 15.1–29.8). Among p‐tau biomarkers, p‐tau231 (17.2%, 95% CI: 17.2–19.7) demonstrated the lowest CV_G_, followed by p‐tau217 (21.1%, 95% CI: 15.1–29.3) and p‐tau181 (25.7%, 95% CI: 18.3–36.1%). Table 3 indicates the CV_G_s separately for males and females. No differences in CV_G_ estimates were found for the evaluated biomarkers, with overlapping 95% CIs between males and females for all measurands. Table 3 also shows the mean concentrations and their 95% CIs for males and females separately, with slightly higher concentrations in females observed for plasma GFAP and NfL, and slightly higher concentrations in males for plasma p‐tau181 and p‐tau231.

### 3.6 Analytical performance specifications and other metrics

Table 4 shows APSs based on the desirable criteria (intermediate stringency), for imprecision, bias. In terms of desirable assay imprecision, the highest demand was for plasma Aβ42/Aβ40 (CV_APS_ < 1.7%), with the lowest demands for plasma p‐tau217 (CV_APS_ < 5.2%) and p‐tau181 (CV_APS_ < 8.4%). Table 4 shows the estimated RCVs, as well as the number of samples needed to estimate the homeostatic point. Plasma Aβ42/Aβ40 demonstrated the lowest RCVs needed for a significant decrease (11%) and for an increase (13%). Similar RCVs for both decrease (−20% to –28%) and increase (26% to 38%) were observed for GFAP, NfL, p‐tau217, and p‐tau231, with the highest RCVs for p‐tau181 (decrease: −38.3%; increase: 62.2%).

**TABLE 4:** Metrics derived from BV estimates.

	Analytical performance specifications		Reference change value, %			Serial samples needed to estimate each individual's homeostatic setpoint within varying proximities to the true value		
	Imprecision, % CV_APS_	Bias, % B_APS_	Decrease	Increase	Index of individuality	± 5%	± 10%	± 20%
Aβ42	3.25	8.31	–17.76	21.60	0.46	8	2	1
Aβ40	3.25	7.06	–17.57	21.31	0.56	7	2	1
Aβ42/Aβ40	1.66	3.69	–11.54	13.04	0.68	3	1	1
GFAP	4.00	15.59	–24.60	32.62	0.34	16	4	1
NfL	3.69	11.21	–23.49	30.71	0.43	15	4	1
P‐tau181	8.36	15.34	–38.34	62.18	0.69	48	12	3
P‐tau217	5.17	11.73	–27.71	38.33	0.56	21	5	1
P‐tau231	3.17	9.17	–20.70	26.10	0.49	11	3	1

*Note*: The table displays each biomarker's desirable analytical performance specifications (APS) for replicate precision and bias. Also, the table displays the reference change values (RCV), which indicate the percentage change needed between two consecutive measurements so that such an increase or decrease significantly overcomes analytical and biological variation. The index of individuality and number of samples needed to estimate the homeostatic point with a given proximity are also shown.

Abbreviations: Aβ, amyloid beta; B_APS,_ bias analytical performance specification; CV_APS_, imprecision analytical performance specification; GFAP, glial fibrillary acidic protein; NfL, neurofilament light; p‐tau, phosphorylated tau.

## 4 DISCUSSION

We report BV estimates for AD plasma biomarkers generated based on a high number of weekly samples per individual, for a comprehensive biomarker panel measured within the same participants. These are the first reported BV estimates for blood Aβ42, Aβ40, Aβ42/Aβ40, and p‐tau, but also for plasma NfL and GFAP (previously evaluated in serum).^55^, ^56^, ^57^ We found that within‐ and between‐subject biological variation can be considerably different for AD biomarker classes, which may impact biomarkers differently according to each application context.

Beyond improving the interpretation of laboratory tests, reliable BV data enable the determination of the APS needed for each biomarker. Assay imprecision (i.e., CV_A_) should be considerably lower than the biomarker's CV_I_, with the desirable analytical performance being that CV_A_ ≤ CV_I_/2.^58^ Here, CV_A_s were slightly higher than desired for most biomarkers. Plasma Aβ42 and Aβ40 (but not Aβ42/Aβ40) and p‐tau181 were within the desirable range, with p‐tau217 showing a very close to desirable analytical performance (CV_A_ = 5.7%; CV_APS_ ≤ 5.2%). Nevertheless, it is important to emphasize that BV estimates are not standalone criteria to determine APS for assays, but rather a complementary tool to refine the determination of analytical goals given each analyte's clinical application context. For instance, the higher‐than‐desirable observed CV_A_s are likely not a cause of concern in light of the main clinical applications of NfL and p‐tau variants, whereas Aβ42/Aβ40 might be more affected, as discussed below.

Plasma p‐tau is an AD‐specific biomarker envisioned to be implemented as a screening tool to classify patients seeking medical advice for cognitive symptoms into high, intermediate, and low risk of having AD pathology.^59^, ^60^ In our study, CV_A_ was remarkably similar for all three plasma p‐tau variants, but they demonstrated considerably different CV_I_s. Interestingly, p‐tau231 demonstrated the lowest CV_I_ (6.3%, 95% CI: 5.5–7.4%), followed by p‐tau217 (10.3%, 95% CI: 9.2–11.7%) and p‐tau181 (16.7%, 95% CI: 12.5%–23.6%), and this could suggest possible differences in release, clearance, or transportation of plasma p‐tau species. Recent head‐to‐head comparisons of plasma p‐tau variants showed the most promising candidates were increased between 100% and 360% in the presence of AD pathology.^9^, ^10^ Considering the magnitude of these increases, currently available p‐tau assays demonstrate satisfactory analytical performance for clinical applications, making their diagnostic ability less vulnerable to biological and analytical variation.

Plasma NfL has been successfully introduced in some clinical routine laboratories, being useful in a range of neurodegenerative diseases and acute neurological conditions.^22^, ^24^, ^26^, ^61^ Plasma NfL showed a relatively low CV_I_ (7.4%, 95% CI: 6.4–8.5%), and a higher CV_G_ (21.2%, 95% CI: 15.1–29.8%). This is in accordance with the ≈ 10% CV_I_ and CV_G_s reported in a previous BV study evaluating serum NfL in a Turkish cohort with 10 weekly collections.^57^ Our RCVs for NfL (increase: +30.7; decrease: −23.4%) also closely agreed with those in that study (increase: +32.7; decrease: −24.7%). A study in a Danish cohort reported a lower CV_I_ for serum NfL with non‐overlapping CIs (CV_I_ = 3%, 95% CI: 1.2–5.0%), and lower RCVs (increase: +24.3; decrease: −19.5%).^56^ These lower estimates could be attributed to the shorter sampling period (three consecutive days vs. 10 weeks), which may underestimate BV.^62^ The relatively high CV_G_ seen for plasma NfL (reflecting higher inter‐individual variability) may pose a challenge to its clinical interpretation in conditions with modest NfL fold changes such as AD, where the diagnostic utility of NfL remains limited. However, in certain clinical scenarios, the magnitude of NfL increases is much larger compared to this higher between‐subject variation, including differentiating primary psychiatric disorders from frontotemporal dementia,^63^ or in prognostic evaluation of acute conditions such as cardiac arrest, stroke, and traumatic brain injury.^24^, ^26^, ^64^

While it is not yet clear what GFAP in the blood reflects (showing unexpected differences against CSF GFAP),^65^ it has been associated with Aβ pathology, showing promising diagnostic performance,^18^, ^20^ even though increased GFAP levels have been reported in Aβ‐negative neurodegenerative conditions.^66^ We observed a relatively low CV_I_ for plasma GFAP (8.0%, 95% CI: 7.0–9.2), and the highest CV_G_ among all biomarkers (30.1%, 95% CI: 21.8–41.6%). A previous study with serum GFAP reported a similar CV_I_ (9.7%, 95% CI: 7.6–11.8), and also a high CV_G_ (39.5%, 95% CI: 31.7–47.3), with RCVs also agreeing between studies. The high between‐individual variability of plasma GFAP, alongside its poorly understood clinical meaning, may pose difficulties for its individual‐level interpretation.

Plasma Aβ42/Aβ40 is a widely investigated biomarker associated with Aβ pathology currently in clinical use to support an AD diagnosis.^12^, ^67^, ^68^ However, in Aβ‐positive versus Aβ‐negative individuals, plasma Aβ42/Aβ40 is only decreased by 8% to 14%.^13^ This clinical context places, per se, an issue for this biomarker, because the modest disease‐related fold changes are in a similar magnitude to that of common analytical variation figures seen in clinical chemistry.^2^, ^14^, ^15^, ^16^ Our BV findings further support that plasma Aβ42/Aβ40 will likely face long‐term difficulties if introduced in clinical practice. Plasma Aβ42/Aβ40 demonstrated a considerably low CV_I_ (3.3%, 95% CI: 2.9–3.9), and a relatively low CV_G_ (6.6%, 95% CI: 4.6–9.3). The low CV_I_ introduces a very high demand on analytical performance, because, desirably, the CV_A_ should be less than half of the CV_I_ (CV_APS_ = 1.7%; Table 4), and, optimally, less or equal to a quarter of CV_I_ (CV_I_/4 = 0.7%). In contrast, plasma p‐tau presents larger disease‐related increases, making the ≈ 5.5% CV_A_s acceptable. Additionally, while there are several different plasma Aβ assays currently in use, they present similarities in their biochemical target and in small disease changes.^13^ Considering previous BV studies showing different assay versions for the same analyte present indistinguishable CV_I_s, it is expected that the CV_I_ for plasma Aβ42/Aβ40 would be similar across assays.^47^, ^52^ Further, the desirable bias is also considerably low for plasma Aβ42/Aβ40 (B_APS_ = 3.7%; Table 4), and it is unlikely that batch‐to‐batch variations could be kept low enough to meet this APS.

We report RCVs for AD plasma biomarkers, which can be potentially clinically valuable when monitoring individuals over time by enumerating the change that can be explained by biological and analytical variation, becoming especially relevant with novel anti‐Aβ immunotherapies such as lecanemab and donanemab.^27^, ^69^ These drugs substantially reduced plasma p‐tau217 levels, on a group level, as early as at 12 weeks of treatment (with our RCVs derived in a similar time frame), during the initial phase of Aβ‐plaque reduction.^27^ RCVs could be potentially used to identify whether a reduction in plasma p‐tau217 after treatment initiation could indeed be related to a positive treatment response. On the other hand, this class of drugs can cause amyloid‐related imaging abnormalities (ARIA) of the hemorrhage or edema type, which can be very harmful.^70^ If NfL proves capable of tracking such changes, RCVs could also be potentially useful to monitor ARIA emergence. However, it is important to consider that our RCVs were obtained using the presumably healthy cognitively unimpaired sample and assays herein described, and, for this reason, cannot be considered universal values, and each laboratory has to determine their own RCVs based on their CV_A_ estimates.

**FIGURE 3:**
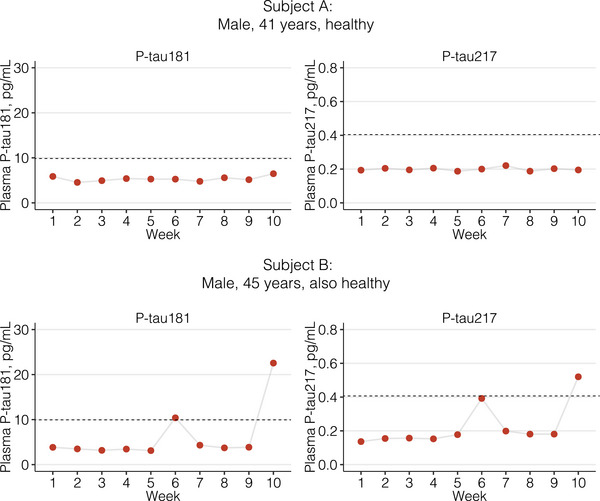
Example of 10‐week variability in plasma p‐tau181 and p‐tau217 in two study subjects. The figure shows the variability in plasma p‐tau181 and p‐tau217 levels over 10 weeks in two study subjects. Dots correspond to the mean concentration of the two duplicate measurements, and all of these subjects’ measurements demonstrated acceptable agreement between replicates, indicating that any deviation observed does not come from analytical imprecision. Dashed lines represent previously published cut‐offs for plasma p‐tau181 and p‐tau217 as illustrative examples of the potential impacts of these fluctuations over decision making. Of note, the outlier data points were excluded from CV_I_ calculations in the homogeneity analyses as they do not reflect the expected homeostatic fluctuation. It is likely that these outlier data points instead correspond to a yet unknown factor that affects biomarker readings. CV_I_, within‐individual biological variation; p‐tau, phosphorylated tau.

Our study provides a unique opportunity to evaluate shorter term fluctuations of AD blood biomarkers. Most published studies have collected samples either cross‐sectionally or over longer periods of time (e.g., 6 months, yearly), and little is known about their shorter term variability. Figure 3 shows plasma p‐tau181 and p‐tau217 for two male subjects with similar ages. Subject “A” shows minimal fluctuation around the homeostatic point, while subject “B” experiences two spikes in plasma p‐tau. These fluctuations were not analytical outliers, and because a chronic disease like AD is unlikely to manifest an oscillatory progression from week to week in middle‐aged adults, there might be yet uncharacterized factors influencing biomarker readings. Clinical decisions made on a single sample collected on a p‐tau “spike” day could erroneously classify patients as “abnormal,” as exemplified by two previously described cut‐offs.^43^, ^71^ Such high‐value outliers are not uncommon in Aβ‐negative groups when examining data points from recent cross‐sectional studies,^4^, ^5^, ^6^ and we recommend caution for researchers and clinicians when interpreting AD blood biomarker results from a single sample. While this has also happened to the other biomarkers in our study, we chose to highlight this phenomenon for p‐tau to demonstrate that even the most promising biomarker classes may be subject to unexpected variations that need to be characterized.

Additionally, knowledge of CV_I_ enables calculating the number of samples needed to estimate the individual's homeostatic point (HSP) within a certain proximity of the true value. To estimate the true HSPs of all analytes with a deviation of ± 20% (α < 0.05), a reasonable margin for most analytes here (given clinical contexts discussed above), one sample suffices for all biomarkers but p‐tau181 (NHSP = 3). Reducing this deviation to 5% (likely needed for plasma Aβ42/Aβ40 but not for others), three samples would be required (Table 4). The II evaluates the utility of reference intervals (RIs). For analytes with pronounced individuality and a relatively low CV_I_ compared to CV_G_ (II < 0.6), RCVs are more useful than RIs for accurate interpretation of sequential results, with each individual serving as the optimal reference point for assessing serial results. However, RIs remain suitable for analytes with high II (particularly when II > 1.4).^33^, ^36^ Here, all II values were below 0.6, except for p‐tau181 and Aβ42/Aβ40, which had slightly higher values, indicating marked individuality for these analytes.^33^, ^36^, ^72^

This study has limitations. Although well powered in individual‐level serial sampling, the number of participants was relatively small, possibly affecting CV_G_ more than CV_I_ estimates. We found some concentration differences between males and females, and an unexpected sex difference in CV_I_ for p‐tau181, warranting further studies. The relatively younger population studied here may not capture biomarker fluctuations related to factors such as co‐morbidities and medication use.^73^ Lack of confirmatory CSF or PET biomarkers prevented us from evaluating the effects of AD pathology over BV estimates. Further evaluation on ethnically diverse populations is also needed. The use of citrate plasma does not affect the obtained CV values, but biomarker concentrations (such as those in Figure 1) should be interpreted bearing in mind the matrix type and collection tube. For most of the evaluated AD biomarkers, citrate plasma has shown very similar concentrations compared to paired ethylenediaminetetraacetic acid, and slightly lower levels in citrate plasma for NfL and GFAP.^44^ Our study has a number of strengths, involving quantifying a comprehensive panel of AD biomarkers in a dataset following all EFLM recommendations for BV studies,^30^, ^31^ which has generated reliable BV data for many other analytes.^37^, ^38^

## CONFLICT OF INTEREST STATEMENT

K.B. has served as a consultant and on advisory boards for Acumen, ALZPath, BioArctic, Biogen, Eisai, Lilly, Moleac Pte. Ltd, Novartis, Ono Pharma, Prothena, Roche Diagnostics, and Siemens Healthineers; has served on data monitoring committees for Julius Clinical and Novartis; has given lectures, produced educational materials, and participated in educational programs for AC Immune, Biogen, Celdara Medical, Eisai, and Roche Diagnostics; and is a co‐founder of Brain Biomarker Solutions in Gothenburg AB (BBS), which is a part of the GU Ventures Incubator Program, outside the work presented in this paper. N.J.A. has given lectures for BioArtic, Eli Lily, and Quanterix. H.Z. has served on scientific advisory boards and/or as a consultant for Abbvie, Acumen, Alector, Alzinova, ALZPath, Annexon, Apellis, Artery Therapeutics, AZTherapies, CogRx, Denali, Eisai, Nervgen, Novo Nordisk, Optoceutics, Passage Bio, Pinteon Therapeutics, Prothena, Red Abbey Labs, reMYND, Roche, Samumed, Siemens Healthineers, Triplet Therapeutics, and Wave; has given lectures in symposia sponsored by Cellectricon, Fujirebio, Alzecure, Biogen, and Roche; and is a co‐founder of Brain Biomarker Solutions in Gothenburg AB (BBS), which is a part of the GU Ventures Incubator Program (outside submitted work). A.J. is employed by ALZPath. All other authors declare no conflicts of interest. Author disclosures are available in the supporting information.

## CONSENT STATEMENT

All volunteers in the EuBIVAS study provided informed consent. The EuBIVAS study received approval from the institutional ethical review board of San Raffaele Hospital, in compliance with the World Medical Association Declaration of Helsinki, as well as the ethical board/regional ethics committee for each participating center.

## Supporting information

Supporting information.
